# Impact of specialist rehabilitation services on hospital length of stay and associated costs

**DOI:** 10.1007/s10198-017-0952-0

**Published:** 2017-12-27

**Authors:** A. Duarte, C. Bojke, W. Cayton, A. Salawu, B. Case, L. Bojke, G. Richardson

**Affiliations:** 1 Centre for Health Economics, University of York, Heslington, York YO10 5DD UK; 2 Faculty of Medicine and Health, Academic Unit of Health Economics, Leeds Institute of Health Sciences, University of Leeds, Leeds, LS2 9NL UK; 3 Hull and East Yorkshire Hospitals NHS Trust, Hull, HU3 2JZ UK; 4 Hull York Medical School, Hull, HU6 7RX UK; 5 Department of Health, Sports and Exercise Science, University of Hull, Hull, HU6 7RX UK; 6 NHS Vale of York Clinical Commissioning Group, York, YO1 6GA UK

**Keywords:** Specialist rehabilitation, Trauma, Complex disability, Length of stay, Local decision making, I (Health Education and Welfare), I0 (General), I1 (Health)

## Abstract

**Background:**

Provision of specialist rehabilitation services in North Yorkshire and Humberside may be suboptimal. Local commissioning bodies need to prioritise investments in health care, but previous studies provide limited evidence to inform the decision to expand existing services on the basis of cost-effectiveness. We examine the impact of specialist rehabilitation services in the subregion on hospital length of stay (LoS) and associated costs compared to routine care.

**Methods:**

Comparison of hospital LoS and associated costs in centres with greater access (Hull) and limited access (i.e. routine care, York and Northern Lincolnshire), to specialist rehabilitation services for patients with complex disabilities following illness or injury, using Hospital Episodes Statistics data.

**Results:**

Average LoS and duration costs by Healthcare Resource Group (HRG) were lower for the majority of patients with greater access to specialist rehabilitation compared to routine care. Difference in LoS between groups widened with level of complexity within each HRG. For the more frequent HRG codes, the LoS difference was as high as 34 days longer for York compared to Hull and £7900 more costly.

**Conclusion:**

Rehabilitation patients within York and Northern Lincolnshire areas appear to have longer LoS and higher associated costs compared to those admitted to the Hull Trust. This analysis suggests that specialist rehabilitation may be cost saving compared to routine care and supports the case for expansion of the existing services to improve coverage in the area.

**Electronic supplementary material:**

The online version of this article (10.1007/s10198-017-0952-0) contains supplementary material, which is available to authorized users.

## Introduction

Specialist rehabilitation provides highly structured multidisciplinary care to individuals with complex disabilities following illness or injury to maximise their recovery after hospital admission and support a safe transition to the community [1]. In the United Kingdom (UK), Clinical Commissioning Groups (CCGs) are responsible for planning and funding rehabilitation services at a local level. However, budget constraints and the numerous competing demands on health and social care impose the need for prioritising investments in health care. This process can be informed by estimates of cost-effectiveness analyses of health interventions or services.

North Yorkshire and Humberside was amongst the regions identified in the latest Major Trauma Peer Review of the UK National Health Service (NHS) to be affected by scarcity of dedicated rehabilitation services for major trauma patients [2]. Although there is currently an inpatient specialist rehabilitation unit within this area, the capacity is limited to 15 beds. This is unlikely to cover the needs of the population (1.5 million), as clinical guidance recommends that 45–65 specialist rehabilitation beds should be available per million population [3]. An evaluation was thus designed to inform the CCGs in this subregion on the impact of expanding the provision of specialist rehabilitation services.

Previous studies suggest that multidisciplinary rehabilitation may reduce length of stay in acute care, reduce continued need for care and improve patient functional outcomes in working age adults with acquired brain injury [4–6]. However, existing studies either lack a control group [4, 5, 7–10] or use inappropriate comparators (e.g. compare levels of rehabilitation intensity or different rehabilitation programmes) [11–14]. This limits their usefulness in informing decisions regarding the provision of specialist rehabilitation services.

There is a lack of robust data on the uptake of rehabilitation services and the costs and benefits implied at CCG level. An alternative approach to demonstrate the value of implementing the proposed rehabilitation services is to use routine data to retrospectively identify patients eligible for specialist rehabilitation and to compare the costs and outcomes between those who access the services and those who do not. The major obstacle is to identify a comparison group of comparable patients who do not access these specialist rehabilitation services.

In this study, we have used routinely collected Hospital Episode Statistics (HES) data in the North Yorkshire and Humberside subregion to compare the inpatient duration of stay and associated costs of patients eligible for specialist rehabilitation to those with restricted access. To our knowledge this is the first study in rehabilitation that uses routinely collected data to inform local decision making in the absence of published evidence that is directly relevant to the decision context.

## Methods

### Aim of the analysis

The analysis aimed to evaluate the impact of the provision of specialist rehabilitation services in three NHS Trusts: Hull and East Yorkshire Hospitals NHS Trust (HEY), York Teaching Hospital NHS Foundation Trust (YRK), and Northern Lincolnshire and Goole NHS Foundation Trust (NLG). These three providers are part of the same subregional Major Trauma Operational Delivery Network (North Yorkshire and Humberside). Castle Hill hospital is part of HEY and hosts a 15-bed unit, where a multidisciplinary team led by a consultant in specialist rehabilitation medicine delivers care to patients. The conditions treated at this unit include traumatic brain injury, brain infections and haemorrhages, brain tumours, myelitis, Guillain–Barre syndrome, neuropathy after critical illness, cerebral palsy, progressive conditions (e.g. multiple sclerosis) and other types of major trauma including amputations. The Castle Hill rehabilitation unit delivers services to a complex caseload in line with local level specialist rehabilitation (2b) [15]. For the purpose of the analysis, YRK and NLG were considered the comparison groups which only have limited specialist rehabilitation available (have no local specialist rehabilitation service and can only access this type of service by being transferred outside of the origin trust area). This mostly limits the access for YRK and NLG to the availability of rehabilitation beds in HEY and can be considered the routine care available for the majority of the area. HEY is the intervention group with greater access to specialist rehabilitation, as it has priority access to the rehabilitation beds at Castle Hill.

### Data utilised for analysis

We extracted data from the cleaned 2013–2014 Admitted Patient Care and the Critical Care components of the Hospital Episode Statistics (HES) data set. HES is a large observational database containing details of all admissions, outpatient appointments and accident and emergency attendances at NHS hospitals in England; it collects data that allows hospitals to be paid for the care delivered, as well as ascertain the quality of care. It is the prime source of data for the provision of hospital services to NHS patients, and it also includes patients treated in the private sector but who are publicly funded. HES data are provided in Finished Consultant Episodes (FCEs), the basic unit of record, [16] which refers to the time a patient spends under the care of one individual consultant in one health care provider. We constructed continuous inpatient spells (CIPS) from these FCEs. CIPS are a continuous period of care within the NHS, regardless of any transfers across providers, and track patients across consultants and/or hospitals as part of their period of care. A CIPS ends when the patient is discharged to home/other location for at least 2 days (or dies). The year of analysis corresponds to the most up-to-date HES data available at the time of the analysis.

### Identification of patients eligible for specialist rehabilitation

The first stage of analysis consisted of the identification of CIPS deemed highly likely to require specialist rehabilitation, for the three providers of interest (HEY, YRK and NLG) from the 2013/14 HES data set. The hospital units (including major trauma centre, trauma units and rehabilitation facilities) covered by these providers compose a subregional Major Trauma Operational Delivery Network. CIPS were extracted if either the spell (i.e. a continuous period of care within the same provider) or FCE Healthcare Resource Group (HRG) contained any of the rehabilitation identifying HRGs or had a primary diagnosis in any of the rehabilitation identifying International Classification of Diseases (ICD) codes. HRGs are standard groupings of clinically similar treatments which use common levels of health care resources [17]. The identifying HRG and ICD codes were selected in collaboration with the CCG and clinical advisors, and were considered to cover the patients with severe illness or injury leading to disability and major trauma requiring specialist rehabilitation. The ICD codes included mainly neurological and spinal conditions, while the HRG codes expanded this to also include admissions related to multiple trauma and amputations. We also included HRG codes specific to rehabilitation services and excluded from the data set any CIPS whose duration was less than 2 days and where the HRG did not appear in the specialist centre treated codes, i.e. those codes for which there were no admissions to Castle Hill hospital. Thus, we only included CIPSs that recorded identifying HRG or ICD codes which had also been recorded for the HEY admissions, resulting in a transfer to Castle Hill hospital where specialist rehabilitation was available. The full list of ICD and HRG codes is shown in the Online Supplement. This enabled us to identify individuals who were potentially eligible for specialist rehabilitation in each of the three centres. The intervention group was composed of patients identified as eligible for specialist rehabilitation services (as described above) initially admitted to HEY and with greater access to these services due to their priority access to the Castle Hill rehabilitation ward. The two comparison groups included patients eligible for specialist rehabilitation who were admitted initially to hospital in (a) YRK or (b) NLG, where access to specialist rehabilitation services is restricted due to the inexistence of these services locally and conditioned by the existence of free rehabilitation beds at Castle Hill hospital. Thus, patients in the comparator groups (YRK and NLG) can only access specialist rehabilitation if they are transferred to Castle Hill hospital and there is sufficient capacity, as the rehabilitation beds would be filled primarily by patients in the intervention group (HEY).

### Attaching unit costs to inpatient stays

Provider level 2013/14 NHS reference costs were used to match costs to each FCE. The excess bed days reference costs were used as measure of per diem costs of stay and were used as the basis of costing the duration of CIPS. The cost of each FCE was obtained by multiplying the unit cost associated with HRG code recorded for the FCE by the length of stay (LoS). Critical care stay was also costed at the trust level reference cost per diem for each CIPS (2013/14). Where an FCE had an associated critical care spell, the associated duration of that critical care period was then deducted from the duration of the FCE so as to avoid double counting. The cost of duration of a CIPS was then calculated as the sum of the adjusted duration costs of each FCE plus the duration costs of critical care. The total cost was estimated by adding any “unbundled’ HRG costs to the CIPS duration cost. The cost of the first FCE was reported, as any resource use occurring on initial admission to hospital is likely to be more intensive and, therefore, it may indicate higher severity of the patient. No additional costs associated with the provision of specialist rehabilitation were included separately, unless the HRG code recorded for the episode referred to rehabilitation (“unbundled” HRG codes starting with the characters VC). However, if specialist rehabilitation services are inherently more expensive to provide for a given HRG then this cost is captured within the trust-level reference costs and therefore directly captured in the analysis either via higher trust-level reference costs applied to LoS and/or via the addition of unbundled HRGs.

The results below compare costs associated with specialist rehabilitation in three Hospital Trusts, on the basis of HRGs. Each HRG code has a common four-character root that shares the same description (Table 1). The fifth character of the code refers to the complexity score (combination of complications and comorbidities) with complexity decreasing from A to Z. As HRGs are defined on the principles of being both clinically meaningful and resource homogeneous [17], then a patient with a specific HRG in York should have, by definition, the same expected cost as a patient with the same HRG in HEY or NLG. As such, adjusting for patient case-mix on the basis of HRGs is a standard and sensible approach.

**Table 1:** HRG codes description and unit costs

HRG Root	Spell HRG description	Complication and comorbidity code^a^	Non-elective stay cost per excess bed day
AA06	Major intracranial procedures except trauma, with brain tumours or cerebral cysts	F	£307
AA12	Intermediate intracranial procedures except trauma, with brain tumours or cerebral cysts	E	£374
AA24	Brain tumours or cerebral cysts	E	£232
		F	£257
		G	£259
		H	£275
AA25	Cerebral degenerations or miscellaneous disorders of nervous system	C	£321
		D	£233
		E	£250
		F	£245
		G	£258
AA26	Muscular, balance, cranial or peripheral nerve disorders, epilepsy or head injury	C	£262
		D	£235
		E	£247
		F	£258
		G	£276
		H	£291
HC01	Extradural spine major 2	B	£254
		C	£359
HC02	Extradural spine major 1	F	£308
HC03	Extradural spine intermediate 2	E	£272
		F	£350
HC04	Extradural spine intermediate 1	F	£310
HC27	Degenerative spinal conditions	F	£261
		G	£273

^a^Complexity level decreasing alphabetically from A to Z

National average unit costs for each of the identified HRG codes [18] are presented alongside the definitions (Table 1).

## Results

We report summary results for the two comparisons: (1) Hull and York (Table 2); (2) Hull and Northern Lincolnshire (Table 3). The tables include HRG codes that were only recorded in the trust with greater access to specialist services (HEY), so as to highlight the full set of HRGs that identified patients eligible for specialist rehabilitation in our study. This may be of interest to readers who would like to apply this framework of analysis to comparisons with other providers.

**Table 2:** Average length of stay and costs by HRG code and Trust (HEY and YRK)

Spell HRG	HEY									YRK								
	*N*	PP CH (%)	1st FCE cost	CIPS duration (days)	CC duration (days)	CIPS duration cost	CC cost	Other costs^a^	Total cost	N	PP CH (%)	1st FCE cost	CIPS duration (days)	CC duration (days)	CIPS duration cost	CC cost	Other costs^a^	Total cost
AA06F	19	5.30	£8408	9.5	1.9	£4153	£1852	£111	£6116									
AA12E	31	3.20	£6301	9.2	1.5	£2357	£1370	£209	£3936									
AA24E/F	34	88.20	£3923	12.6	0	£3029	£0	£543	£3572	6	0.00	£2153	21.5	1.2	£4672	£1552	£169	£6393
AA24G	18	83.30	£2268	6.9	0	£2148	£0	£225	£2373	8	12.50	£1294	23.5	0.3	£4866	£220	£191	£5277
AA24H	14	71.40	£1679	4.6	0.1	£1275	£0	£140	£1415	7	0.00	£1269	13.3	2.3	£3635	£2868	£161	£6664
AA25D	5	40.00	£754	18	0	£4756	£0	£126	£4882									
AA25E/F	39	5.10	£1335	9.5	0.3	£2287	£201	£243	£2731	11	0.00	£1720	17.8	1	£3642	£1306	£50	£4998
AA25G	63	4.80	£1158	5.1	0	£1471	£46	£159	£1676	53	0.00	£851	6.1	0.1	£1602	£80	£91	£1773
AA26C/D/E	28	21.40	£1343	13.7	1.3	£3728	£1991	£107	£5825	11	0.00	£2239	47.5	0.7	£11,642	£600	£119	£12,361
AA26F	59	3.40	£1013	9.5	1.5	£2479	£1697	£97	£4273	19	0.00	£1251	10.8	0.4	£2946	£432	£75	£3453
AA26G	111	3.60	£1064	5.7	0.3	£1605	£247	£95	£1947	82	0.00	£1031	6.3	0.1	£1534	£157	£60	£1751
AA26H	146	4.10	£712	4.7	0	£1460	£35	£108	£1603	163	0.00	£720	5.2	0.1	£1649	£137	£52	£1838
HC01B	8	25.00	£8510	4.1	0	£1012	£0	£0	£1012									
HC01C	22	77.30	£6350	3.5	0	£851	£0	£5	£856									
HC02F	37	13.50	£4299	3.2	0.3	£4255	£238	£13	£4506									
HC03E	15	13.30	£5468	10.2	0	£3168	£0	£61	£3229									
HC03F	182	11.50	£3781	3.5	0	£1064	£10	£18	£1092									
HC04E/F	197	17.30	£3485	3	0	£879	£0	£26	£904	3	Redacted given the small number of observations							
HC27F/G	46	4.40	£1275	4.6	0	£1471	£0	£130	£1601	38	2.60	£1021	7.448	0	£1945	£0	£186	£2131

*CC* critical care, *N* number of CIPSs, *PP CH* proportion of CIPSs in Castle Hill Hospital

^a^Refers to “unbundled” HRG costs, i.e. elements of cost and activity that have been “unbundled” from core HRG codes, and are processed separately from the FCE costs. These include elements of cost related to diagnostics, high-cost treatments and drugs. Unbundling allows the core HRGs to be better representative of activity and costs, while still including these potentially high-cost elements separately in the total costs estimation

**Table 3:** Average length of stay and costs by HRG code and Trust (HEY and NLG)

Spell HRG	HEY									NLG								
	N	PP CH (%)	1st FCE cost	CIPS duration (days)	CC duration (days)	CIPS duration cost	CC cost	Other costs^a^	Total cost	*N*	PP CH (%)	1st FCE cost	CIPS duration (days)	CC duration (days)	CIPS duration cost	CC cost	Other costs^a^	Total cost
AA06F	19	5.30	£8408	9.5	1.9	£4153	£1852	£111	£6116									
AA12E	31	3.20	£6301	9.2	1.5	£2357	£1370	£209	£3936									
AA24E/F/G	52	86.50	£3350	10.6	0	£2724	£0	£433	£3157	9	11.10	£1122	11.4	0.2	£4140	£196	£211	£4547
AA24H	14	71.40	£1679	4.6	0.1	£1275	£0	£140	£1415	8	0.00	£1101	12	1.3	£5228	£1099	£349	£6676
AA25C/D	5	40.00	£754	18	0	£4756	£0	£126	£4882	8	0.00	£5225	84.1	3.4	£23,165	£4278	£155	£27,598
AA25F	29	6.90	£1207	9.5	0	£2401	£0	£245	£2646	16	0.00	£978	7.8	0.3	£2860	£226	£166	£3252
AA25G	63	4.80	£1158	5.1	0	£1471	£46	£159	£1676	62	3.20	£966	7	0.5	£2309	£603	£128	£3040
AA26C/D/E	28	21.40	£1343	13.7	1.3	£3728	£1991	£107	£5825	9	0.00	£1682	36.3	1.8	£10,024	£1526	£156	£11,706
AA26F	59	3.40	£1013	9.5	1.5	£2479	£1697	£97	£4273	18	0.00	£698	8.1	0.5	£2481	£689	£128	£3298
AA26G	111	3.60	£1064	5.7	0.3	£1605	£247	£95	£1947	76	0.00	£670	5.7	0.7	£1688	£653	£87	£2428
AA26H	146	4.10	£712	4.7	0	£1460	£35	£108	£1603	121	0.00	£475	4.8	0.3	£1617	£207	£89	£1913
HC01B	8	25.00	£8510	4.1	0	£1012	£0	£0	£1012									
HC01C	22	77.30	£6350	3.5	0	£851	£0	£5	£856									
HC02F	37	13.50	£4299	3.2	0.3	£4255	£238	£13	£4506									
HC03E	15	13.30	£5468	10.2	0	£3168	£0	£61	£3229									
HC03F	182	11.50	£3781	3.5	0	£1064	£10	£18	£1092									
HC04E	23	8.70	£4570	5	0	£1397	£0	£39	£1436									
HC04F	174	18.40	£3342	2.8	0	£810	£0	£24	£834									
HC27F/G	46	4.40	£1275	4.6	0	£1471	£0	£130	£1601	15	0.00	£997	6.667	0	£1948	£0	£182	£2130

*CC* critical care, *N* number of CIPS, *PP CH* proportion of CIPS in Castle Hill Hospital

^a^Refers to “unbundled” HRG costs, i.e. elements of cost and activity that have been “unbundled” from core HRG codes, and are processed separately from the FCE costs. These include elements of cost related to diagnostics, high cost treatments and drugs. Unbundling allows the core HRGs to be better representative of activity and costs, while still including these potentially high cost elements separately in the total costs estimation

### Comparison between Hull and York

Within each HRG four character code root, the increase in complexity score (from Z to A) is accompanied by an increase in length of stay (CIPS duration), cost of the first FCE of the CIPS and CIPS duration costs (Table 2). This is as expected; patients who have more complex needs would have longer admissions and be more costly to manage.

The majority of patients in YRK had a longer LoS and a greater CIPS duration cost than patients in corresponding HRG groups in HEY (Table 2). The differences in first FCE costs between HEY and YRK (across HRG codes) are less noticeable, suggesting that the case-mix in the two trusts are comparable. LoS in critical care was generally short and differences between the two trusts small, but this translated into differences in mean costs per HRG code of a greater magnitude. This is reflective of the type of care provided in these units, which includes more treatments and therapies and hence is very resource intensive and more costly to the NHS compared to a stay in a general ward.

For the HRG roots with a greater volume of CIPS, the differences in CIPS duration and total costs appears to widen with the complexity of the patients (Table 2). For example, within HRG root AA26 (Muscular, Balance, Cranial or Peripheral Nerve Disorders, Epilepsy or Head Injury) patients classified under the more complex code in YRK have a CIPS duration 34 days greater on average than in HEY with duration costs approximately £7900 higher. At the lowest complexity level reported for this HRG root, the difference in average CIPS duration is similar in YRK and HEY. However, the numbers of CIPSs for greater complexity levels are small (even after aggregation within HRG roots), so we would expect these values to be less robust than for HRGs with more CIPSs. Similar trends can be observed for the HRG codes AA24 and AA25.

As the length of stay and duration costs are greater in YRK than in Hull across the majority HRG codes, this may indicate that patients are being retained longer in inpatient services at YRK given the lack of a local specialist rehabilitation service, thus driving costs upwards in YRK.

### Comparison between Hull and Northern Lincolnshire

Similar to the comparison between HEY and YRK Trusts, the patterns of increased CIPS duration, cost of the first FCE of the CIPS and total costs with increased complexity score are also observed for the HEY and NLG (Table 3). Similarly to YRK, a small percentage of patients initially admitted to the Northern Lincolnshire hospitals are transferred to Castle Hill.

The overall length of stay and total costs, as well as time spent in critical care and respective costs, are greater in NLG than in HEY, for the majority of HRG codes. This suggests that patients might be retained longer in inpatient services in Northern Lincolnshire. Similarly to YRK this appears to be the driver of higher costs in NLG compared to HEY.

The most representative HRG code roots with CIPSs in NLG are the same as for HEY and YRK. Across these codes, the more sizeable differences in CIPS duration and total costs between Hull and Northern Lincolnshire are again driven by the codes indicating higher complexity. For example, the aggregate HRG code AA26C/D/E (a higher complexity code than the other codes sharing this AA26 root in Table 3); in NLG the CIPS duration is 23 days greater on average than in HEY and £6296 more costly.

The results suggest that between the three centres the more complex cases have lower CIPS duration and associated costs in the centre with specialist rehabilitation (HEY) than the other two centres (YRK and NLG). Therefore, the provision of specialist rehabilitation facilities may, on the basis of this analysis, reduce downstream costs compared to routine care.

## Discussion

In general, patients eligible for specialist rehabilitation within the YRK and NLG areas have longer length of stays and higher duration costs compared to a similar group of patients admitted to HEY.

This study presents the first evaluation of local level specialised inpatient rehabilitation services in the United Kingdom that compares these services with the care that is provided in their absence. It highlights potential cost savings of investing in specialist rehabilitation services. This study uses high quality local level routinely collected data to compare the cost of providing specialist rehabilitation services with the cost of providing usual care. Although the number of cases identified at local level is small and not amenable to formal statistical analysis, the study suggests that there might be cost savings associated with the provision of specialist rehabilitation care compared to routine care. Therefore, these results are directly relevant for informing the commissioners of the proposed services.

The analysis presented above has some limitations. It is usual in economic evaluation to consider both costs and benefits; the analysis above only considers costs, given the limitations highlighted above of available measures of benefits. Nevertheless, the level of cost saving associated with provision of specialist care suggests that unless that specialist care is associated with a detrimental effect on patients’ health outcomes, specialist rehabilitation is likely to be cost-effective. It is worth noting that there is some evidence suggesting that specialist rehabilitation may improve patients’ functional outcomes, as well as reduce the need for care in a community setting [4–6]. The results of these studies could not be combined with our analyses, due to methodological limitations that are likely to generate biased estimates of costs and benefits. However, it suggests that specialist rehabilitation may also improve health outcomes and reduce weekly hours of care after discharge, even if the size of that benefit is uncertain.

Another potential limitation is that the analysis relies on the assumption that the intervention (HEY) and comparator groups (YRK and NLG) are correctly identified. While we can never be certain that this is the case, the HRG and ICD codes used to identify the groups were validated by clinical advisors who considered the identification strategy a reasonable approach.

A further limitation is the small number of observations, which may limit the robustness of the results. Importantly, the small number of observations precludes the use of statistical methods such as propensity score matching to adjust for confounding variables that may simultaneously impact on costs and likelihood of using the rehabilitation services. Examples of such confounders are age, sex, existence of comorbidities, clinical severity/complexity of underlying condition, etc. We have presented the results by spell HRG code, so that differences in complexity could be made evident, and also estimated the cost of the first FCE (which should reflect complexity). By comparing at HRG code level and analysing differences in FCE costs we can reduce the impact of any confounding at complexity level, when analysing the differences between groups. We conducted robustness checks on the data, examining the distribution of total costs (Tables C and D, Online Supplement) and the potential impact of outliers on results (Tables E and F, Online supplement). Despite the exclusion of high cost outliers from the analysis, greater access to specialist rehabilitation still appears to be associated with lower mean total costs compared to limited access to these services. However, we cannot exclude the possibility that the differences in LoS and associated costs between the two trusts are driven by factors other than the availability of a specialist inpatient rehabilitation service.

Finally, the analysis does not reflect local capacity constraints in providing such a service. There is an implicit assumption that beds can be generated using existing capacity. This is true of most evaluations and guidelines based on these evaluations.

This analysis supports the case for expansion of specialist rehabilitation services and suggests that clinical guidance advising a higher level of specialist rehabilitation input for these conditions should be considered.

## Electronic supplementary material

Below is the link to the electronic supplementary material.

Supplementary material 1 (DOCX 39 kb)

## Abbreviations

CCG: Clinical Commissioning Group
CIPS: Continuous inpatient spells
FCE: Finished Consultant Episode
HES: Hospital Episode Statistics
HEY: Hull and East Yorkshire Hospitals NHS Trust
HRG: Healthcare Resource Group
ICD: International Classification of Diseases
LoS: Length of stay
NHS: National Health Service
NLG: Northern Lincolnshire and Goole NHS Foundation Trust
UK: United Kingdom
YRK: York Teaching Hospital NHS Foundation Trust
