# Supplementary material for: Impact of specialist rehabilitation services on hospital length of stay and associated costs
Source: Eur J Health Econ. 2017 Dec 27;19(7):1027–34. doi: 10.1007/s10198-017-0952-0 (PMC6105206; doi:10.1007/s10198-017-0952-0)
Supplement: Supplementary file 1 — Supplementary material 1 (DOCX 39 kb) [file 10198_2017_952_MOESM1_ESM.docx]

Online Supplement

Table A ICD codes identifying patients potentially eligible for inpatient specialist rehabilitation services

| ICD code | Description |
| --- | --- |
| C70.0 | Malignant neoplasm: Cerebral meninges |
| C70.1 | Malignant neoplasm: Spinal meninges |
| C70.9 | Malignant neoplasm: Meninges, unspecified |
| C71.0 | Malignant neoplasm: Cerebrum, except lobes and ventricles |
| C71.1 | Malignant neoplasm: Frontal lobe |
| C71.2 | Malignant neoplasm: Temporal lobe |
| C71.3 | Malignant neoplasm: Parietal lobe |
| C71.4 | Malignant neoplasm: Occipital lobe |
| C71.5 | Malignant neoplasm: Cerebral ventricle |
| C71.6 | Malignant neoplasm: Cerebellum |
| C71.7 | Malignant neoplasm: Brain stem |
| C71.8 | Malignant neoplasm: Overlapping lesion of brain |
| C71.9 | Malignant neoplasm: Brain, unspecified |
| C72.0 | Malignant neoplasm: Spinal cord |
| C72.1 | Malignant neoplasm: Cauda equina |
| C72.2 | Malignant neoplasm: Olfactory nerve |
| C72.3 | Malignant neoplasm: Optic nerve |
| C72.4 | Malignant neoplasm: Acoustic nerve |
| C72.5 | Malignant neoplasm: Other and unspecified cranial nerves |
| C72.8 | Malignant neoplasm: Overlapping lesion of brain and other parts of central nervous system |
| C72.9 | Malignant neoplasm: Central nervous system, unspecified |
| C73.X | Malignant neoplasm of thyroid gland |
| C74.0 | Malignant neoplasm: Cortex of adrenal gland |
| C74.1 | Malignant neoplasm: Medulla of adrenal gland |
| C74.9 | Malignant neoplasm: Adrenal gland, unspecified |
| C75.0 | Malignant neoplasm: Parathyroid gland |
| C75.1 | Malignant neoplasm: Pituitary gland |
| C75.2 | Malignant neoplasm: Craniopharyngeal duct |
| C75.3 | Malignant neoplasm: Pineal gland |
| D32.0 | Benign neoplasm: Cerebral meninges |
| D32.1 | Benign neoplasm: Spinal meninges |
| D32.9 | Benign neoplasm: Meninges, unspecified |
| D33.0 | Benign neoplasm: Brain, supratentorial |
| D33.1 | Benign neoplasm: Brain, infratentorial |
| D33.2 | Benign neoplasm: Brain, unspecified |
| D33.3 | Benign neoplasm: Cranial nerves |
| D33.4 | Benign neoplasm: Spinal cord |
| D33.7 | Benign neoplasm: Other specified parts of central nervous system |
| D33.9 | Benign neoplasm: Central nervous system, unspecified |
| D35.4 | Benign neoplasm: Pineal gland |
| G06.0 | Intracranial abscess and granuloma |
| G06.1 | Intraspinal abscess and granuloma |
| G06.2 | Extradural and subdural abscess, unspecified |
| G07.X | Intracranial and intraspinal abscess and granuloma in diseases classified elsewhere |
| G08.X | Intracranial and intraspinal phlebitis and thrombophlebitis |
| G09.X | Sequelae of inflammatory diseases of central nervous system |
| G11.1 | Early-onset cerebellar ataxia |
| G11.2 | Late-onset cerebellar ataxia |
| G11.3 | Cerebellar ataxia with defective DNA repair |
| G11.4 | Hereditary spastic paraplegia |
| G11.8 | Other hereditary ataxias |
| G11.9 | Hereditary ataxia, unspecified |
| G12.1 | Other inherited spinal muscular atrophy |
| G12.2 | Motor neuron disease |
| G12.8 | Other spinal muscular atrophies and related syndromes |
| G12.9 | Spinal muscular atrophy, unspecified |
| G13.0 | Paraneoplastic neuromyopathy and neuropathy |
| G13.1 | Other systemic atrophy primarily affecting central nervous system in neoplastic disease |
| G13.2 | Systemic atrophy primarily affecting central nervous system in myxoedema |
| G13.8 | Systemic atrophy primarily affecting central nervous system in other diseases classified elsewhere |
| G14.X | Postpolio syndrome |
| G21.1 | Other drug-induced secondary parkinsonism |
| G23.2 | Striatonigral degeneration |
| G23.8 | Other specified degenerative diseases of basal ganglia |
| G23.9 | Degenerative disease of basal ganglia, unspecified |
| G24.0 | Drug-induced dystonia |
| G24.1 | Idiopathic familial dystonia |
| G24.2 | Idiopathic nonfamilial dystonia |
| G24.3 | Spasmodic torticollis |
| G24.4 | Idiopathic orofacial dystonia |
| G57.8 | Other mononeuropathies of lower limb |
| G57.9 | Mononeuropathy of lower limb, unspecified |
| G58.0 | Intercostal neuropathy |
| G58.7 | Mononeuritis multiplex |
| G58.8 | Other specified mononeuropathies |
| G58.9 | Mononeuropathy, unspecified |
| G59.0 | Diabetic mononeuropathy |
| G59.8 | Other mononeuropathies in diseases classified elsewhere |
| G60.0 | Hereditary motor and sensory neuropathy |
| G60.1 | Refsum's disease |
| G60.2 | Neuropathy in association with hereditary ataxia |
| G60.3 | Idiopathic progressive neuropathy |
| G60.8 | Other hereditary and idiopathic neuropathies |
| G60.9 | Hereditary and idiopathic neuropathy, unspecified |
| G61.0 | Guillain-Barré syndrome |
| G61.1 | Serum neuropathy |
| G61.8 | Other inflammatory polyneuropathies |
| G61.9 | Inflammatory polyneuropathy, unspecified |
| G62.0 | Drug-induced polyneuropathy |
| G62.1 | Alcoholic polyneuropathy |
| G62.2 | Polyneuropathy due to other toxic agents |
| G62.8 | Other specified polyneuropathies |
| G62.9 | Polyneuropathy, unspecified |
| G63.0 | Polyneuropathy in infectious and parasitic diseases classified elsewhere |
| G63.1 | Polyneuropathy in neoplastic disease |
| G63.2 | Diabetic polyneuropathy |
| G63.3 | Polyneuropathy in other endocrine and metabolic diseases |
| G63.4 | Polyneuropathy in nutritional deficiency |
| G63.5 | Polyneuropathy in systemic connective tissue disorders |
| G63.6 | Polyneuropathy in other musculoskeletal disorders |
| G63.8 | Polyneuropathy in other diseases classified elsewhere |
| G64.X | Other disorders of peripheral nervous system |
| G70.0 | Myasthenia gravis |
| G70.1 | Toxic myoneural disorders |
| G70.2 | Congenital and developmental myasthenia |
| G70.8 | Other specified myoneural disorders |
| G70.9 | Myoneural disorder, unspecified |
| G71.0 | Muscular dystrophy |
| G71.1 | Myotonic disorders |
| G71.2 | Congenital myopathies |
| G71.3 | Mitochondrial myopathy, not elsewhere classified |
| G71.8 | Other primary disorders of muscles |
| G71.9 | Primary disorder of muscle, unspecified |
| G72.0 | Drug-induced myopathy |
| G72.1 | Alcoholic myopathy |
| G72.2 | Myopathy due to other toxic agents |
| G72.3 | Periodic paralysis |
| G72.4 | Inflammatory myopathy, not elsewhere classified |
| G72.8 | Other specified myopathies |
| G72.9 | Myopathy, unspecified |
| G73.0 | Myasthenic syndromes in endocrine diseases |
| G73.1 | Lambert-Eaton syndrome |
| G73.2 | Other myasthenic syndromes in neoplastic disease |
| G73.4 | Myopathy in infectious and parasitic diseases classified elsewhere |
| G73.5 | Myopathy in endocrine diseases |
| G73.6 | Myopathy in metabolic diseases |
| G73.7 | Myopathy in other diseases classified elsewhere |
| G80.0 | Spastic quadriplegic cerebral palsy |
| G80.1 | Spastic diplegic cerebral palsy |
| G80.2 | Spastic hemiplegic cerebral palsy |
| G80.3 | Dyskinetic cerebral palsy |
| G80.4 | Ataxic cerebral palsy |
| G80.8 | Other cerebral palsy |
| G80.9 | Cerebral palsy, unspecified |
| G81.0 | Flaccid hemiplegia |
| G81.1 | Spastic hemiplegia |
| G81.9 | Hemiplegia, unspecified |
| G82.0 | Flaccid paraplegia |
| G82.1 | Spastic paraplegia |
| G82.2 | Paraplegia, unspecified |
| G82.3 | Flaccid tetraplegia |
| G82.4 | Spastic tetraplegia |
| G82.5 | Tetraplegia, unspecified |
| G83.0 | Diplegia of upper limbs |
| G83.1 | Monoplegia of lower limb |
| G83.2 | Monoplegia of upper limb |
| G83.3 | Monoplegia, unspecified |
| G83.4 | Cauda equina syndrome |
| G83.8 | Other specified paralytic syndromes |
| G83.9 | Paralytic syndrome, unspecified |
| G90.0 | Idiopathic peripheral autonomic neuropathy |
| G90.1 | Familial dysautonomia [Riley-Day] |
| G90.2 | Horner's syndrome |
| G90.3 | Multi-system degeneration |
| G90.4 | Autonomic dysreflexia |
| G90.8 | Other disorders of autonomic nervous system |
| G90.9 | Disorder of autonomic nervous system, unspecified |
| G91.0 | Communicating hydrocephalus |
| G91.1 | Obstructive hydrocephalus |
| G91.2 | Normal-pressure hydrocephalus |
| G91.3 | Post-traumatic hydrocephalus, unspecified |
| G91.8 | Other hydrocephalus |
| G91.9 | Hydrocephalus, unspecified |
| G92.X | Toxic encephalopathy |
| G93.0 | Cerebral cysts |
| G93.1 | Anoxic brain damage, not elsewhere classified |
| G93.2 | Benign intracranial hypertension |
| G93.3 | Postviral fatigue syndrome |
| G93.4 | Encephalopathy, unspecified |
| G93.5 | Compression of brain |
| G93.6 | Cerebral oedema |
| G93.7 | Reye's syndrome |
| G93.8 | Other specified disorders of brain |
| G93.9 | Disorder of brain, unspecified |
| G94.0 | Hydrocephalus in infectious and parasitic diseases classified elsewhere |
| G94.1 | Hydrocephalus in neoplastic disease |
| G94.2 | Hydrocephalus in other diseases classified elsewhere |
| G94.8 | Other specified disorders of brain in diseases classified elsewhere |
| G95.0 | Syringomyelia and syringobulbia |
| G95.1 | Vascular myelopathies |
| G95.2 | Cord compression, unspecified |
| G95.8 | Other specified diseases of spinal cord |
| G95.9 | Disease of spinal cord, unspecified |
| G96.0 | Cerebrospinal fluid leak |
| G96.1 | Disorders of meninges, not elsewhere classified |
| G96.8 | Other specified disorders of central nervous system |
| G96.9 | Disorder of central nervous system, unspecified |
| G97.0 | Cerebrospinal fluid leak from spinal puncture |
| G97.1 | Other reaction to spinal and lumbar puncture |
| G97.2 | Intracranial hypotension following ventricular shunting |
| G97.8 | Other postprocedural disorders of nervous system |
| G97.9 | Postprocedural disorder of nervous system, unspecified |
| G98.X | Other disorders of nervous system, not elsewhere classified |
| G99.0 | Autonomic neuropathy in endocrine and metabolic diseases |
| G99.1 | Other disorders of autonomic nervous system in other diseases classified elsewhere |
| G99.2 | Myelopathy in diseases classified elsewhere |
| G99.8 | Other specified disorders of nervous system in diseases classified elsewhere |
| M46.8 | Other specified inflammatory spondylopathies |
| M46.9 | Inflammatory spondylopathy, unspecified |
| M47.0 | Anterior spinal and vertebral artery compression syndromes |
| M47.1 | Other spondylosis with myelopathy |
| M47.2 | Other spondylosis with radiculopathy |
| M47.8 | Other spondylosis |
| M47.9 | Spondylosis, unspecified |
| M48.0 | Spinal stenosis |
| M48.1 | Ankylosing hyperostosis [Forestier] |
| M48.2 | Kissing spine |
| M48.3 | Traumatic spondylopathy |
| M48.4 | Fatigue fracture of vertebra |
| M48.5 | Collapsed vertebra, not elsewhere classified |
| M48.8 | Other specified spondylopathies |
| M48.9 | Spondylopathy, unspecified |
| M49.0 | Tuberculosis of spine |
| M49.1 | Brucella spondylitis |
| M49.2 | Enterobacterial spondylitis |
| M49.3 | Spondylopathy in other infectious and parasitic diseases classified elsewhere |
| M49.4 | Neuropathic spondylopathy |
| M49.5 | Collapsed vertebra in diseases classified elsewhere |
| M49.8 | Spondylopathy in other diseases classified elsewhere |
| M50.0 | Cervical disc disorder with myelopathy |
| M50.1 | Cervical disc disorder with radiculopathy |
| M50.2 | Other cervical disc displacement |
| M50.3 | Other cervical disc degeneration |
| M50.8 | Other cervical disc disorders |
| M50.9 | Cervical disc disorder, unspecified |
| M51.0 | Lumbar and other intervertebral disc disorders with myelopathy |
| M51.1 | Lumbar and other intervertebral disc disorders with radiculopathy |
| M51.2 | Other specified intervertebral disc displacement |
| M51.3 | Other specified intervertebral disc degeneration |
| M51.4 | Schmorl's nodes |
| M51.8 | Other specified intervertebral disc disorders |
| M51.9 | Intervertebral disc disorder, unspecified |
| M53.0 | Cervicocranial syndrome |
| M53.1 | Cervicobrachial syndrome |
| M53.2 | Spinal instabilities |
| M53.3 | Sacrococcygeal disorders, not elsewhere classified |
| M53.8 | Other specified dorsopathies |
| M53.9 | Dorsopathy, unspecified |

Table B – HRG codes identifying patients potentially eligible for inpatient specialist rehabilitation services

Table B HRG codes identifying patients potentially eligible for inpatient specialist rehabilitation services

| **HRG Currency Code** | **Currency Description** |
| --- | --- |
| AA25C | Cerebral Degenerations or Miscellaneous Disorders of Nervous System, with CC Score 14+ |
| AA25D | Cerebral Degenerations or Miscellaneous Disorders of Nervous System, with CC Score 11-13 |
| AA25E | Cerebral Degenerations or Miscellaneous Disorders of Nervous System, with CC Score 8-10 |
| AA25F | Cerebral Degenerations or Miscellaneous Disorders of Nervous System, with CC Score 5-7 |
| AA25G | Cerebral Degenerations or Miscellaneous Disorders of Nervous System, with CC Score 0-4 |
| AA26C | Muscular, Balance, Cranial or Peripheral Nerve Disorders, Epilepsy or Head Injury, with CC Score 15+ |
| AA26D | Muscular, Balance, Cranial or Peripheral Nerve Disorders, Epilepsy or Head Injury, with CC Score 12-14 |
| AA26E | Muscular, Balance, Cranial or Peripheral Nerve Disorders, Epilepsy or Head Injury, with CC Score 9-11 |
| AA26F | Muscular, Balance, Cranial or Peripheral Nerve Disorders, Epilepsy or Head Injury, with CC Score 6-8 |
| AA26G | Muscular, Balance, Cranial or Peripheral Nerve Disorders, Epilepsy or Head Injury, with CC Score 3-5 |
| AA26H | Muscular, Balance, Cranial or Peripheral Nerve Disorders, Epilepsy or Head Injury, with CC Score 0-2 |
| AA28C | Motor Neuron Disease with CC Score 8+ |
| AA28D | Motor Neuron Disease with CC Score 5-7 |
| AA28E | Motor Neuron Disease with CC Score 2-4 |
| AA28F | Motor Neuron Disease with CC Score 0-1 |
| AA50A | Very Complex Intracranial Procedures, 19 years and over, with CC Score 12+ |
| AA50B | Very Complex Intracranial Procedures, 19 years and over, with CC Score 6-11 |
| AA50C | Very Complex Intracranial Procedures, 19 years and over, with CC Score 0-5 |
| AA50D | Very Complex Intracranial Procedures, 18 years and under, with CC Score 12+ |
| AA50E | Very Complex Intracranial Procedures, 18 years and under, with CC Score 6-11 |
| AA50F | Very Complex Intracranial Procedures, 18 years and under, with CC Score 0-5 |
| AA51A | Complex Intracranial Procedures, 19 years and over, with CC Score 12+ |
| AA51B | Complex Intracranial Procedures, 19 years and over, with CC Score 8-11 |
| AA51C | Complex Intracranial Procedures, 19 years and over, with CC Score 4-7 |
| AA51D | Complex Intracranial Procedures, 19 years and over, with CC Score 0-3 |
| AA51E | Complex Intracranial Procedures, 18 years and under, with CC Score 8+ |
| AA51F | Complex Intracranial Procedures, 18 years and under, with CC Score 4-7 |
| AA51G | Complex Intracranial Procedures, 18 years and under, with CC Score 0-3 |
| AA52A | Very Major Intracranial Procedures, 19 years and over, with CC Score 12+ |
| AA52B | Very Major Intracranial Procedures, 19 years and over, with CC Score 8-11 |
| AA52C | Very Major Intracranial Procedures, 19 years and over, with CC Score 4-7 |
| AA52D | Very Major Intracranial Procedures, 19 years and over, with CC Score 0-3 |
| AA52E | Very Major Intracranial Procedures, 18 years and under, with CC Score 8+ |
| AA52F | Very Major Intracranial Procedures, 18 years and under, with CC Score 4-7 |
| AA52G | Very Major Intracranial Procedures, 18 years and under, with CC Score 0-3 |
| AA53A | Major Intracranial Procedures, 19 years and over, with CC Score 12+ |
| AA53B | Major Intracranial Procedures, 19 years and over, with CC Score 8-11 |
| AA53C | Major Intracranial Procedures, 19 years and over, with CC Score 4-7 |
| AA53D | Major Intracranial Procedures, 19 years and over, with CC Score 0-3 |
| AA53E | Major Intracranial Procedures, 18 years and under, with CC Score 8+ |
| AA53F | Major Intracranial Procedures, 18 years and under, with CC Score 4-7 |
| AA53G | Major Intracranial Procedures, 18 years and under, with CC Score 0-3 |
| HC20H | Vertebral Column Injury with Interventions |
| HC20J | Vertebral Column Injury without Interventions, with CC Score 6+ |
| HC20K | Vertebral Column Injury without Interventions, with CC Score 3-5 |
| HC27H | Degenerative Spinal Conditions with Interventions, with CC Score 3+ |
| HC27K | Degenerative Spinal Conditions without Interventions, with CC Score 9+ |
| HC27L | Degenerative Spinal Conditions without Interventions, with CC Score 6-8 |
| HC27M | Degenerative Spinal Conditions without Interventions, with CC Score 3-5 |
| HC28H | Spinal Cord Conditions with Interventions |
| HC28J | Spinal Cord Conditions without Interventions, with CC Score 7+ |
| HC28K | Spinal Cord Conditions without Interventions, with CC Score 5-6 |
| HC28L | Spinal Cord Conditions without Interventions, with CC Score 3-4 |
| HC31H | Spinal Infection with Interventions, with CC Score 6+ |
| HC50A | Very Complex Instrumented Correction of Spinal Deformity, 19 years and over |
| HC51A | Complex Instrumented Correction of Spinal Deformity, 19 years and over, with CC Score 7+ |
| HC51B | Complex Instrumented Correction of Spinal Deformity, 19 years and over, with CC Score 3-6 |
| HC52A | Complex Spinal Reconstructive Procedures with CC Score 7+ |
| HC52B | Complex Spinal Reconstructive Procedures with CC Score 4-6 |
| HC53A | Very Major Spinal Reconstructive Procedures with CC Score 4+ |
| HC53B | Very Major Spinal Reconstructive Procedures with CC Score 2-3 |
| HC53C | Very Major Spinal Reconstructive Procedures with CC Score 0-1 |
| HC54A | Major Spinal Reconstructive Procedures with CC Score 4+ |
| HC54B | Major Spinal Reconstructive Procedures with CC Score 2-3 |
| HC54C | Major Spinal Reconstructive Procedures with CC Score 0-1 |
| HC60A | Very Complex Extradural Spinal Procedures with CC Score 4+ |
| HC60B | Very Complex Extradural Spinal Procedures with CC Score 2-3 |
| HC60C | Very Complex Extradural Spinal Procedures with CC Score 0-1 |
| HC61A | Complex Extradural Spinal Procedures with CC Score 4+ |
| HC61B | Complex Extradural Spinal Procedures with CC Score 2-3 |
| HC61C | Complex Extradural Spinal Procedures with CC Score 0-1 |
| HC62A | Very Major Extradural Spinal Procedures with CC Score 4+ |
| HC62B | Very Major Extradural Spinal Procedures with CC Score 2-3 |
| HC62C | Very Major Extradural Spinal Procedures with CC Score 0-1 |
| HC63A | Major Extradural Spinal Procedures with CC Score 4+ |
| HC63B | Major Extradural Spinal Procedures with CC Score 2-3 |
| HC63C | Major Extradural Spinal Procedures with CC Score 0-1 |
| HC71Z | Major Intradural Spinal Procedures |
| VA13A | Multiple Trauma with Diagnosis Score <=23, with Intervention Score 19-29 |
| VA13B | Multiple Trauma with Diagnosis Score 24-32, with Intervention Score 19-29 |
| VA13C | Multiple Trauma with Diagnosis Score 33-50, with Intervention Score 19-29 |
| VA13D | Multiple Trauma with Diagnosis Score >=51, with Intervention Score 19-29 |
| VA14A | Multiple Trauma with Diagnosis Score <=23, with Intervention Score 30-44 |
| VA14B | Multiple Trauma with Diagnosis Score 24-32, with Intervention Score 30-44 |
| VA14C | Multiple Trauma with Diagnosis Score 33-50, with Intervention Score 30-44 |
| VA14D | Multiple Trauma with Diagnosis Score >=51, with Intervention Score 30-44 |
| VA15A | Multiple Trauma with Diagnosis Score <=23, with Intervention Score >=45 |
| VA15B | Multiple Trauma with Diagnosis Score 24-32, with Intervention Score >=45 |
| VA15C | Multiple Trauma with Diagnosis Score 33-50, with Intervention Score >=45 |
| VA15D | Multiple Trauma with Diagnosis Score >=51, with Intervention Score >=45 |
| YQ20A | Amputation of Multiple Limbs with CC Score 10+ |
| YQ21A | Amputation of Single Limb with Other Blood Vessel Procedure, with CC Score 10+ |
| YQ22A | Amputation of Single Limb with CC Score 10+ |
| VC06Z | Rehabilitation for brain injuries |
| VC08Z | Rehabilitation for Spinal Cord injuries |
| VC10Z | Rehabilitation for pain syndromes |
| VC12Z | Rehabilitation for other Neurological disorders |
| VC14Z | Rehabilitation for amputation of limb |
| VC16Z | Rehabilitation for hip fracture |
| VC18Z | Rehabilitation for joint replacement |
| VC20Z | Rehabilitation for Inflammatory Arthritis |
| VC22Z | Rehabilitation for Non-inflammatory Arthritis |
| VC24Z | Rehabilitation for other Musculoskeletal disorders |
| VC26Z | Rehabilitation for Drug or Alcohol Addiction |
| VC28Z | Rehabilitation for other Psychiatric disorders |
| VC30Z | Rehabilitation for burns |
| VC32Z | Rehabilitation following Head and Neck Reconstructive Surgery |
| VC34Z | Rehabilitation following other reconstructive surgery |
| VC36Z | Rehabilitation for other trauma |
| VC38Z | Rehabilitation for Acute Myocardial Infarction or Other Cardiac Disorders |
| VC40Z | Rehabilitation for Respiratory disorders |
| VC42Z | Rehabilitation for other disorders |

Table C Total costs by HRG code and Trust (HEY and YRK)

|  | **HEY** | | | | | **YRK** | | | | |
| --- | --- | --- | --- | --- | --- | --- | --- | --- | --- | --- |
|  |  | **Total costs** | | | |  | **Total costs** | | | |
| **Spell HRG** | **N** | **Mean** | **Median** | **Range^*^** | **SD** | **N** | **Mean** | **Median** | **Range^*^** | **SD** |
| AA06F | 19 | £6,117 | £5,846 | £7,836 | £2,381 |  |  |  |  |  |
| AA12E | 31 | £3,936 | £3,963 | £9,008 | £2,041 |  |  |  |  |  |
| AA24E/F | 34 | £3,572 | £2,534 | £20,604 | £3,880 | 6 | £6,393 | £3,015 | £17,748 | £7,273 |
| AA24G | 18 | £2,373 | £1,666 | £7,624 | £2,051 | 8 | £5,277 | £4,415 | £9,245 | £3,490 |
| AA24H | 14 | £1,415 | £1,110 | £2,718 | £863 | 7 | £6,664 | £2,395 | £18,135 | £7,006 |
| AA25D | 5 | £4,882 | £1,960 | £16,061 | £6,860 |  |  |  |  |  |
| AA25E/F | 39 | £2,731 | £1,763 | £15,696 | £2,822 | 11 | £4,998 | £3,466 | £24,352 | £7,026 |
| AA25G | 63 | £1,676 | £1,151 | £10,076 | £1,471 | 53 | £1,773 | £1,020 | £11,214 | £2,042 |
| AA26C/D/E | 28 | £5,825 | £2,373 | £45,527 | £9,006 | 11 | £12,361 | £4,128 | £57,965 | £18,111 |
| AA26F | 59 | £4,274 | £1,601 | £59,924 | £9,297 | 19 | £3,453 | £2,508 | £11,021 | £3,250 |
| AA26G | 111 | £1,947 | £1,228 | £13,815 | £2,152 | 82 | £1,751 | £1,056 | £8,088 | £1,730 |
| AA26H | 146 | £1,602 | £909 | £53,948 | £4,499 | 163 | £1,837 | £1,058 | £54,081 | £4,481 |
| HC01B | 8 | £1,012 | £988 | £988 | £415 |  |  |  |  |  |
| HC01C | 22 | £856 | £779 | £1,759 | £397 |  |  |  |  |  |
| HC02F | 37 | £4,506 | £3,442 | £13,792 | £2,545 |  |  |  |  |  |
| HC03E | 15 | £3,229 | £617 | £21,869 | £6,646 |  |  |  |  |  |
| HC03F | 182 | £1,092 | £633 | £32,135 | £2,575 |  |  |  |  |  |
| HC04E/F | 197 | £904 | £639 | £8,906 | £919 | 3 | **Redacted given the small number of observations** | | | |
| HC27F/G | 46 | £1,601 | £969 | £19,195 | £2,834 | 38 | £2,131 | £1,243 | £10,166 | £2,214 |

^*^Difference between the maximum and minimum value; SD, standard deviation.

Table D *Total costs by HRG code and Trust (HEY and NLG)*

|  | **HEY** | | | | | **NLG** | | | | |
| --- | --- | --- | --- | --- | --- | --- | --- | --- | --- | --- |
|  |  | **Total costs** | | | |  | **Total costs** | | | |
| **Spell HRG** | **N** | **Mean** | **Median** | **Range^*^** | **SD** | **N** | **Mean** | **Median** | **Range^*^** | **SD** |
| AA06F | 19 | £6,117 | £5,846 | £7,836 | £2,381 |  |  |  |  |  |
| AA12E | 31 | £3,936 | £3,963 | £9,008 | £2,041 |  |  |  |  |  |
| AA24E/F/G | 52 | £3,157 | £2,143 | £20,604 | £3,387 | 9 | £4,547 | £2,933 | £10,363 | £4,032 |
| AA24H | 14 | £1,415 | £1,110 | £2,718 | £863 | 8 | £6,676 | £7,480 | £10,339 | £4,000 |
| AA25C/D | 6 | £5,756 | £2,435 | £16,061 | £6,498 | 8 | £27,598 | £7,924 | £93,174 | £40,702 |
| AA25F | 29 | £2,646 | £1,641 | £15,696 | £3,177 | 16 | £3,253 | £1,648 | £7,624 | £2,739 |
| AA25G | 63 | £1,676 | £1,151 | £10,076 | £1,471 | 62 | £3,040 | £1,783 | £15,459 | £3,297 |
| AA26C/D/E | 28 | £5,825 | £2,373 | £45,527 | £9,006 | 9 | £11,706 | £6,320 | £47,034 | £14,468 |
| AA26E | 59 | £4,274 | £1,601 | £59,924 | £9,297 | 18 | £3,298 | £1,434 | £12,769 | £3,802 |
| AA26G | 111 | £1,947 | £1,228 | £13,815 | £2,152 | 76 | £2,427 | £1,365 | £32,881 | £4,002 |
| AA26H | 146 | £1,602 | £909 | £53,948 | £4,499 | 121 | £1,912 | £1,275 | £19,175 | £2,516 |
| HC01B | 8 | £1,012 | £988 | £988 | £415 |  |  |  |  |  |
| HC01C | 22 | £856 | £779 | £1,759 | £397 |  |  |  |  |  |
| HC02F | 37 | £4,506 | £3,442 | £13,792 | £2,545 |  |  |  |  |  |
| HC03E | 15 | £3,229 | £617 | £21,869 | £6,646 |  |  |  |  |  |
| HC03F | 182 | £1,092 | £633 | £32,135 | £2,575 |  |  |  |  |  |
| HC04E | 23 | £1,436 | £567 | £8,838 | £2,300 |  |  |  |  |  |
| HC04F | 174 | £834 | £639 | £2,997 | £492 |  |  |  |  |  |
| HC27F/G | 46 | £1,601 | £969 | £19,195 | £2,834 | 15 | £2,129 | £1,505 | £6,905 | £1,690 |

^*^Difference between the maximum and minimum value; SD, standard deviation.

Table E Average total costs by HRG code and Trust (HEY and YRK) after the removal of outliers

|  | **HEY** | | | | | | **YRK** | | | | | |
| --- | --- | --- | --- | --- | --- | --- | --- | --- | --- | --- | --- | --- |
|  |  |  | **Total costs** | | | |  |  | **Total costs** | | | |
| **Spell HRG** | **N_out_** | **N- N_out_^*^** | **Mean** | **Median** | **Range^**^** | **SD** | **N** | **N- N_out_^*^** | **Mean** | **Median** | **Range^**^** | **SD** |
| AA24E/F | 32 | 2 | £2,743 | £2,245 | £6,214 | £1,664 | 5 | 1 | £4,002 | £2,704 | £11,784 | £4,822 |
| AA24G | 16 | 2 | £1,779 | £1,557 | £4,264 | £1,078 | 8 | 0 | £5,277 | £4,415 | £9,245 | £3,490 |
| AA24H | 12 | 2 | £1,107 | £1,023 | £1,182 | £391 | 6 | 1 | £4,657 | £2,144 | £11,907 | £5,007 |
| AA25E/F | 37 | 2 | £2,200 | £1,717 | £4,493 | £1,389 | 10 | 1 | £3,016 | £2,294 | £6,748 | £2,614 |
| AA25G | 60 | 3 | £1,428 | £1,138 | £3,372 | £751 | 50 | 3 | £1,362 | £1,010 | £3,533 | £1,015 |
| AA26C/D/E | 27 | 1 | £4,335 | £2,131 | £14,058 | £4,428 | 10 | 1 | £7,742 | £3,783 | £32,501 | £10,183 |
| AA26F | 56 | 3 | £2,352 | £1,576 | £14,560 | £2,381 | 17 | 2 | £2,519 | £1,834 | £6,272 | £1,756 |
| AA26G | 104 | 7 | £1,504 | £1,169 | £4,759 | £1,032 | 77 | 5 | £1,399 | £967 | £4,024 | £1,018 |
| AA26H | 145 | 1 | £1,239 | £909 | £8,434 | £1,001 | 161 | 2 | £1,425 | £1,057 | £9,509 | £1,285 |
| HC27F/G | 45 | 1 | £1,200 | £965 | £4,087 | £815 | 35 | 3 | £1,553 | £1,132 | £3,065 | £907 |

^*^Number of excluded outliers;^**^Difference between the maximum and minimum value; N_out_, Number of observations left in each HRG code after the removal of outliers defined as observations for which the total costs were greater than the median total costs plus two standard deviations for that HRG code; SD, standard deviation.

Table F Average total costs by HRG code and Trust (HEY and NLG) after the removal of outliers

|  | **HEY** | | | | | | **NLG** | | | | | |
| --- | --- | --- | --- | --- | --- | --- | --- | --- | --- | --- | --- | --- |
|  |  |  | **Total costs** | | | |  |  | **Total costs** | | | |
| **Spell HRG** | **N_out_** | **N- N_out_^*^** | **Mean** | **Median** | **Range^**^** | **SD** | **N** | **N- N_out_^*^** | **Mean** | **Median** | **Range^**^** | **SD** |
| AA24E/F/G | 50 | 2 | £2,610 | £2,063 | £7,881 | £1,801 | 7 | 2 | £2,548 | £2,598 | £2,139 | £789 |
| AA24H | 12 | 2 | £1,107 | £1,023 | £1,182 | £391 | 8 | 0 | £6,676 | £7,480 | £10,339 | £4,000 |
| AA25C/D | 5 | 1 | £3,489 | £1,960 | £9,096 | £3,775 | 6 | 2 | £5,762 | £3,080 | £12,594 | £5,509 |
| AA25F | 27 | 2 | £1,912 | £1,332 | £4,358 | £1,272 | 14 | 2 | £2,521 | £1,512 | £6,095 | £2,010 |
| AA25G | 60 | 3 | £1,428 | £1,138 | £3,372 | £751 | 57 | 5 | £2,209 | £1,689 | £7,731 | £1,604 |
| AA26C/D/E | 27 | 1 | £4,335 | £2,131 | £14,058 | £4,428 | 8 | 1 | £7,155 | £6,026 | £16,393 | £5,120 |
| AA26E | 56 | 3 | £2,352 | £1,576 | £14,560 | £2,381 | 16 | 2 | £2,157 | £1,408 | £7,919 | £1,941 |
| AA26G | 104 | 7 | £1,504 | £1,169 | £4,759 | £1,032 | 75 | 1 | £2,015 | £1,359 | £7,980 | £1,769 |
| AA26H | 145 | 1 | £1,239 | £909 | £8,434 | £1,001 | 119 | 2 | £1,620 | £1,275 | £5,616 | £1,113 |
| HC27F/G | 45 | 1 | £1,200 | £965 | £4,087 | £815 | 14 | 1 | £1,736 | £1,472 | £2,441 | £755 |

^*^Number of excluded outliers;^**^Difference between the maximum and minimum value; N_out_, Number of observations left in each HRG code after the removal of outliers defined as observations for which the total costs were greater than the median total costs plus two standard deviations for that HRG code; SD, standard deviation.
